# Effects of developmental plasticity on heat tolerance may be mediated by changes in cell size in *Drosophila melanogaster*

**DOI:** 10.1111/1744-7917.12742

**Published:** 2020-01-17

**Authors:** Nadja Verspagen, Félix P. Leiva, Irene M. Janssen, Wilco C. E. P. Verberk

**Affiliations:** ^1^ Department of Animal Ecology and Physiology, Institute for Water and Wetland Research Radboud University Nijmegen The Netherlands; ^2^ Department of Human Genetics, Radboud University Medical Center Radboud Institute for Molecular Life Sciences Nijmegen The Netherlands

**Keywords:** cell size, heat stress, rearing temperature, thermal death time, thermal tolerance

## Abstract

There is a growing interest in the physiology underpinning heat tolerance of ectotherms and their responses to the ongoing rise in temperature. However, there is no consensus about the underlying physiological mechanisms. According to “the maintain aerobic scope and regulate oxygen supply” hypothesis, responses to warming at different organizational levels contribute to the ability to safeguard energy metabolism *via* aerobic pathways. At the cellular level, a decrease in cell size increases the capacity for the uptake of resources (e.g., food and oxygen), but the maintenance of electrochemical gradients across cellular membranes implies greater energetic costs in small cells. In this study, we investigated how different rearing temperatures affected cell size and heat tolerance in the fruit fly *Drosophila melanogaster*. We tested the hypothesis that smaller‐celled flies are more tolerant to acute, intense heat stress whereas larger‐celled flies are more tolerant to chronic, mild heat stress. We used the thermal tolerance landscape framework, which incorporates the intensity and duration of thermal challenge. Rearing temperatures strongly affected both cell size and survival times. We found different effects of developmental plasticity on tolerance to either chronic or acute heat stress. Warm‐reared flies had both smaller cells and exhibited higher survival times under acute, intense heat stress when compared to cold‐reared flies. However, under chronic, mild heat stress, the situation was reversed and cold‐reared flies, consisting of larger cells, showed better survival. These differences in heat tolerance could have resulted from direct effects of rearing temperature or they may be mediated by the correlated changes in cell size. Notably, our results are consistent with the idea that a smaller cell size may confer tolerance to acute temperatures via enhanced oxygen supply, while a larger cell may confer greater tolerance to chronic and less intense heat stress via more efficient use of resources.

## Introduction

In addition to shifts in phenology (Cohen *et al*., 2018) and range shifts (Parmesan & Yohe, 2003), a reduction in body size constitutes the third universal response to the ongoing rise in temperature (Atkinson, 1994; Gardner *et al*., 2011). At higher rearing temperatures, ectotherms grow and develop faster, yet mature at a smaller size, a pattern that has been termed the temperature‐size rule (Atkinson, 1995). Although this pattern is widely observed across arthropods (Atkinson, 1994), there is no consensus on the underlying mechanisms to explain this pattern nor whether it is adaptive to grow smaller in warmer conditions (Angilletta & Dunham, 2003; Kingsolver & Huey, 2008; Chown & Gaston, 2010). A central role for oxygen has been proposed to explain the temperature‐size rule (Atkinson, 1995; Atkinson *et al*., 2006; Verberk & Atkinson, 2013; Hoefnagel & Verberk, 2015; Walczyńska *et al*., 2015).

According to the maintain aerobic scope and regulate oxygen supply hypothesis proposed by Atkinson *et al*. (2006), responses to temperature at different organizational levels (mitochondria, cells, organs and whole organism) contribute to safeguarding the production of energy at higher temperatures *via* aerobic pathways. At these temperatures more energy is required for protein synthesis and for maintaining ionic gradients (Clarke & Fraser, 2004). Decreasing cell size may facilitate oxygen provisioning to the mitochondria and safeguard aerobic energy metabolism, because small cells have relatively larger membrane surface area across which oxygen can diffuse when compared to large cells (Szarski, 1983). Studies have indeed demonstrated that not only does the body size of organisms decrease at higher rearing temperatures, there are also decreases in the size of their cells (Azevedo *et al*., 2002; Czarnołęski *et al*., 2013). Although not all cell‐types respond in the same direction (Blanckenhorn & Llaurens, 2005; Atkinson *et al*., 2006; Czarnołęski *et al*., 2015), a decrease in cell size may partially explain the temperature‐size dependence, as body size is a function of both cell size and cell number (Partridge *et al*., 1994; Hessen *et al*., 2013). While small cells may constitute an advantage in terms of oxygen supply, the downside of having small cells, and consequently a higher membrane surface area to volume ratio, relates to the higher energetic costs needed to maintain electrochemical gradients (Gupta *et al*., 1978). In short, larger cells are more efficient with respect to their energetic metabolism (but see Malerba *et al*., 2018). Thus, the cell size of an organism may reflect a balance between a high capacity for resource uptake (e.g., oxygen) and high energetic costs to maintain ionic gradients across their cellular membranes, an idea which is central to the optimal cell size theory (Szarski, 1983; Kozłowski *et al*., 2003; Czarnołęski *et al*., 2015).

Scaling up in the biological organization, heat tolerance limits have also been linked to insufficient capacity for aerobic metabolism, but limitations at the whole‐organism level are typically being emphasized in the context of heat tolerance (Pörtner, 2001). In this study, we investigated how different rearing conditions (notably rearing temperatures) affected both cell size and heat tolerance in the fruit fly *Drosophila melanogaster*. Rearing temperatures are known to alter both cell size and body size, whereas larval density can affect body size in *D. melanogaster* (Miller & Thomas, 1958; Santos *et al*., 1994).

Heat tolerance is often measured by recording the temperature at which the animal collapses during a ramping up of environmental temperature (*CT*_max_, critical thermal maximum) but several methodological aspects (e.g., ramping rate, starting temperature) are known to be important in modulating *CT*_max_ (Terblanche *et al*., 2007) as they change the duration of exposure to heat stress (Rezende *et al*., 2014). Recent studies have emphasized the importance of accounting for differences in duration of exposure (Leiva *et al*., 2019) and have explicitly included time as a factor (Castañeda *et al*., 2015; Semsar‐Kazerouni & Verberk, 2018; Truebano *et al*., 2018). Here, we followed this approach and incorporated time as an experimental factor by measuring the survival time as a function of varying intensities of heat stress. The interaction of the intensity and duration of thermal stress can be described by using thermal death time curves (Stumbo, 1965; Wang *et al*., 2007), and implemented under the thermal tolerance landscapes framework (Rezende *et al*., 2014). We expected (i) warm‐reared flies to have smaller cells compared to cold‐reared flies, and (ii) warm‐reared flies to survive longer at a high intensity of heat stress than cold‐reared flies. However, the higher energetic costs associated with small cells may cause reductions in performance at longer timescales, and we therefore also expected (iii) better survival of cold‐reared flies at the longer trials conducted at milder stress temperatures.

## Materials and methods

### Stocks origin

*Drosophila melanogaster* were obtained from the Department of Human Genetics of the Radboud UMC (Nijmegen, the Netherlands). In order to reduce the variability attributed to the origin of the different stocks, the vials were pooled and transferred, using the same number of adult flies, to new vials with fresh food. Flies were reared under controlled conditions inside a climate‐controlled room (MC785‐KLIMA, VDH Product BV, Roden, the Netherlands), with temperature (25 °C), photoperiod (light : dark = 12 : 12) and humidity (70%, HOBO® temp/RH logger, Onset Computer Corporation, Bourne, MA, USA) in plastic vials (25 × 95 mm) with about 20 mm of cornmeal‐agar‐yeast food medium for approximately 15 generations before the start of experiments. Flies were pooled at each generation and transferred to vials with fresh food roughly every 12 d.

### Experimental design

Contrasts in cell size were induced both for females and males by rearing the flies at four constant rearing temperatures (17, 21, 25 and 29 °C). To collect the eggs, parental adult flies belonging to 10–15 vials previously maintained at 25 °C, were allowed to lay eggs at 25 °C on agar‐plates for *ca*. 15–20 h overnight. To improve the egg laying, yeast extracts were added to the agar‐plates and several scratches were carved into the agar layer (Markow & O'Grady, 2005). This way, a large enough number of eggs to use in the following experiments was ensured. After this time period, adult flies were removed and the eggs were gently taken out together with a small piece of agar‐medium and then transferred to a new vial with fresh food. The larval density was controlled by adding 40–60 eggs *per* vial (low density) and 80–100 eggs *per* vial (high density) to new vials with fresh food and the exact number of eggs *per* vial was recorded. Preliminary analyses showed no effects of larval density on either cell size or wing size (Tables S1 and S2). Rearing density did affect cell number at the highest rearing temperatures but the effect size was very small (for details, see Fig. S1). For the remainder of the paper we therefore pooled the high and low‐density treatments for each rearing temperature. For each rearing temperature, at least 16 vials were used in order to secure a large enough number of adult flies for cell size measurements (160 adult flies) and thermal tolerance (640 adult flies) experiments. Flies from different rearing temperature were allowed to develop for one generation under controlled conditions of photoperiod (light : dark = 12 : 12) and relative humidity (70%). Only virgin flies, sorted within the first 8 h after eclosion, were used for the experiments. The ranges of the developmental times, from eggs to adult, were 19–23, 12–14, 9–11, and 8–10 d for 17, 21, 25, and 29 °C reared flies, respectively, matching the development times reported in Overgaard *et al*. (2008). In addition, except for males reared at 21 °C, eggs‐to‐adult viability ranged from 65% to 90%, similar to those values reported in the literature (Borash *et al*., 2000). Thus, flies that were reared at different temperatures most likely developed normally and no negative effect of rearing temperature on performance under normal conditions is expected.

### Cell and wing size

**Fig. 1:**
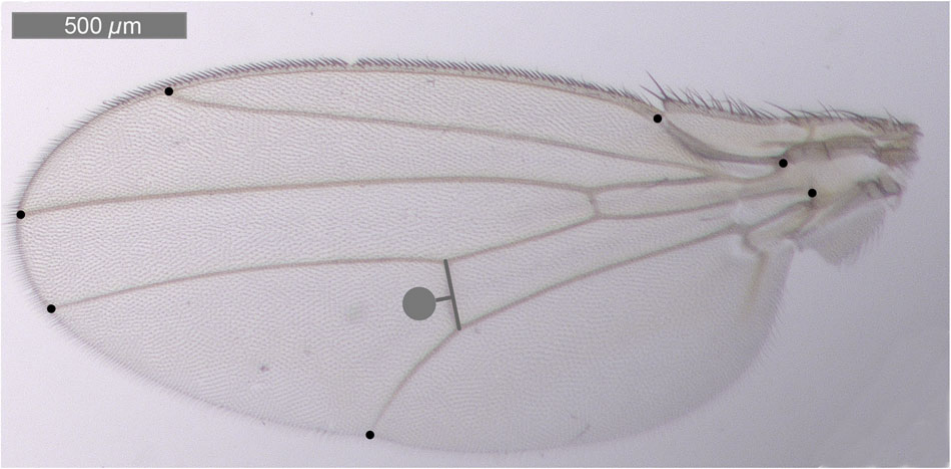
*Drosophila melanogaster* left wing with seven landmarks (black dots) defining the polygon to determinate the wing size. The gray circular area (*ca*. 7580 *µ*m^2^) is the region used for trichome counting and it is superimposed at a 100 *µ*m distance and perpendicular, to the posterior crossvein.

Left wings of 5‐d‐old adult flies in each sex and rearing temperature were used to determine the cell size, cell number, and wing size. The wings were carefully separated from the body under a dissection stereomicroscope with tweezers and mounted on a slide with glycerol: ethanol solution drops. Wing images were captured at two optical magnification (20× and 40×) and using a Leica DM‐RBE FL4 microscope mounting a Leica DFC450 C camera (Wetzlar, Germany). The wing size was measured as the centroid size limited by seven landmarks estimated from the 20× magnified picture using ImageJ (Abràmoff *et al*., 2004). The number of trichomes in a 100 *µ*m diameter‐circular area (*ca*. 7580 *µ*m^2^) was used to calculate the cell area, as each cell secretes a individual trichome (Dobzhansky, 1929). All the trichomes whose base was positioned inside the circle were counted. The counting area was superposed at a 100 *µ*m distance and placed perpendicular to the posterior crossvein on the 40×‐magnified picture (Fig. 1). The cell area was then calculated by dividing the area of the circle by the number of trichomes counted. The cell number *per* wing was calculated by dividing the centroid wing size by the cell area.

### Thermal death times

To determinate the survival time of adult fruit flies under heat‐induced stress, we used thermal death times curves described in the theoretical thermal tolerance landscapes framework (Rezende *et al*., 2014). For this, 30 virgin flies of 7–9 d old for each sex and rearing temperature treatment were individually allocated into capped 4‐mL glass vials and exposed to four static temperatures of 36, 37, 38, and 39 °C which are considered to be stressful for this species (Crill *et al*., 1996). Forty glass vials *per* run were mounted on a Plexiglas™ rack which was immersed in a 9.5‐L glass‐aquarium (125 × 250 × 310 mm) filled with water maintained a constant temperature of 36, 37, 38, or 39 °C. A small pump was used to continuously exchange the water from a programmable digital heating circulating bath (Grant TXF200, Grant Instruments, UK) to the aquarium. During the trials, we measured the water temperature frequently (every 10 min) at the top and at the bottom of the aquarium using a wireless thermometer (Omega HH806AU, Omega Engineering, Inc. Stamford, CT, USA). For each run we used a JVC Camcorder (Everio HD, model number GZHM650BE, JVCKENWOOD Corporation, Yokohama, Japan) to take pictures at a frame rate of 12 frames per minute (i.e., every 5 s). From the picture compilation, we created a reverse video to simplify the survival time analysis (Castañeda *et al*., 2019).

Generally, flies moved around in the vials actively while alive. Therefore, death times were determined as the point at which flies stopped moving. We applied objective criteria to ascertain this endpoint. As a rule, each fly had to move at least twice its own body size in horizontal direction or at least once its own body size in vertical direction to be considered alive. This proved to work well in the vast majority of cases but some individuals did not show such movements but were still clearly alive (making small movements over a prolonged period of time). In these cases, flies were considered to be alive when small movements were observed for at least 5 frames (25 s) in a row.

The flies were left in the aquarium until all individuals stopped moving. To make sure this endpoint was reached, the rack (with vials) was gently shaken. When flies no longer responded to this perturbation, the experiment was stopped. The survival times were determined by two observers who analyzed the videos independently from each other. When two measurements had a difference of more than 5% of the maximum survival time, the survival times of those individuals were determined again by both observers together to reach a final conclusion. Discrepancies between observers were in almost all cases related to viewing or typing errors and thus, faulty values could be identified and corrected upon the third inspection. In the end, the observations of both observers matched closely (*R* ^2^_adj_ = 0.99) and the median survival time between observers was used.

### Statistical analyses

Our data analyses were based on versions of linear models. Within each set of dependent variables (cell size, cell number, wing size, and survival time), we selected the most informative models using a multimodel inference approach by means of the highest Akaike's weights (*w_i_*), which provide the relative weight of the evidence toward one of the all tested models (thus the weights of all the models add up to 1) (Burnham & Anderson, 2002). For cell size, cell number, and wing size models, the rearing temperature was included as a numerical variable and sex (female/male) as a categorical one. The set of models that we compared for these three dependent variables included the full model (sex × rearing temperature) and all possible models without any interactions.

The survival time *t*, on a log_10_ scale, was regressed against stress temperature (in °C) in order to create thermal death time curves. From these curves, we calculated the thermal sensitivity *z*, defined as the temperature change (°C) required for a 10‐fold change in survival time and the static *CT*_max_, defined as the temperature (°C) where the survival time is log_10_ *t =* 0 after 1 min of exposure to high temperatures. We measured *t* and controlled for the temperature at which flies were exposed, resulting relatively straightforward to calculate each one of the two parameters before mentioned as z=−1/slope and *CT*_max_ =−intercept/slope). In our analyses, we tested whether survival time was affected by rearing temperature, sex and cell area. Here, we compared models that were relevant in the context of our research question. The set of models that we compared included the full model, and simplified versions thereof, which included stress temperature or stress temperature and its interactions with one or two other variables (rearing temperature, sex or cell area). As it was not possible to determine the cell area on the same flies used in the thermal death time curves assays, we used the median cell area values previously measured for each of the four rearing condition combinations (note we used the median rather than mean cell area as it was most informative to explain survival time; Table S3). For each response variable, model assumptions (normality and homoscedasticity) were tested in full models with all interactions. No significant departures from normality or homoscedasticity were found throughout the different models applied. As an additional step, we performed variation partitioning analysis in order to estimate the pure and overlaid effects of sex, rearing temperatures and cell area on the survival time. For this, we used the *R* ^2^_adj_ values of the single as well as from combined models to determinate the relative contribution of each explanatory variable in terms of explained variance. All analyses and figures presented in the paper were implemented and generated in R version 3.5.1 (R Development Core Team, 2018) by using the packages “AICcmodavg” (Mazerolle, 2019), “modEvA” (Barbosa *et al*., 2016) and “visreg” (Breheny & Burchett, 2017).

## Results

### Cell size, cell number, and wing size

**Fig. 2:**
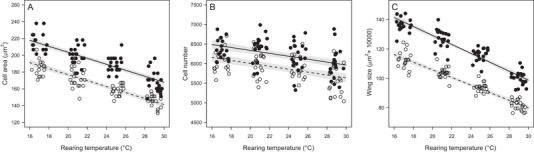
Effects of rearing temperature on cell area (A), cell number (B), and wing size (C) for females (black circles) and males (open circles) of *Drosophila melanogaster*. Solid (for females), dotted (for males) lines and 95% confidence intervals (light shaded areas) represent the regression models with the highest support showed in Tables 1 and 2.

Rearing fruit flies *Drosophila melanogaster* at different temperatures yielded contrasts in cell area which were best explained by additive effects of rearing temperature and sex (Table 1). Cell area was significantly reduced in males (*F*_1,148_ = 244.32; *P* < 0.001, Table 2 and Fig. 2A) and in flies reared at higher temperatures (*F*_1,148_ = 345.99; *P* < 0.001, Table 2 and Fig. 2A) which together accounted for 80% of the variation in cell size (Table 1). Differences in cell number largely mirror those in cell size, with males having fewer cells than females and cell number declining with rearing temperature (Fig. 2B). In contrast, variation in cell number was poorly explained, with the best model explaining only 32% of the variation (Table 1). Variation in wing size was best explained by a model including interaction between sex and rearing temperature, which could account for 92% of the variation. Variation in cell size contributed more strongly to variation in wing size than did variation in cell number (Fig. 2C and Table S4).

**Table 1:** Selection results of linear models to explain variation in the cell area, cell number, and wing size in *Drosophila melanogaster* as a function of sex and rearing temperature (RT)

Response	Effects	*k*	*R* ^2^_adj_	AICc	ΔAICc	*w_i_*	*LL*
Cell area	**Sex + RT**	**4**	**0.80**	**1142.22**	**0.00**	**0.54**	**−566.97**
	**Sex × RT**	**5**	**0.80**	**1142.58**	**0.36**	**0.46**	**−566.08**
	RT	3	0.46	1287.31	145.09	0.00	−640.57
	Sex	3	0.32	1322.11	179.89	0.00	−657.97
Cell number	**Sex + RT**	**4**	**0.32**	**2192.63**	**0.00**	**0.73**	**−1092.18**
	Sex × RT	5	0.31	2194.63	2.00	0.27	−1092.11
	RT	3	0.16	2223.45	30.82	0.00	−1108.64
	Sex	3	0.16	2223.45	30.82	0.00	−1108.64
Wing size	**Sex × RT**	**5**	**0.92**	**3687.79**	**0.00**	**0.91**	**−1838.69**
	Sex + RT	4	0.92	3692.34	4.55	0.09	−1842.03
	RT	3	0.52	3966.04	278.25	0.00	−1979.94
	Sex	3	0.40	3999.44	311.65	0.00	−1996.64

The number of parameters (*k*), the goodness of fit (*R* ^2^_adj_), corrected Akaike's information criterion (AICc), the difference in AICc value respect to the best model ΔAICc, Akaike's weights (*w_i_*), and log‐likelihood (*LL*) are indicated for each model. Bold indicates models with the ΔAICc < 2.

**Table 2:** Outcomes of linear models with the ΔAICc < 2 for cell area, cell number and wing size highlighted in Table 1. Degrees of freedom (df), sum of squares (SS), Fisher statistic (*F*), and probability values (*P*) are indicated for each model

Model	Source	df	SS	*F*	*P*‐value
Cell area ∼ Sex + RT	Sex	1	26 641	244.32	<0.001
	RT	1	37 728	345.99	<0.001
	Residuals	148	16 138		
Cell area ∼ Sex × RT	Sex	1	26 641	245.55	<0.001
	RT	1	37 728	347.73	<0.001
	Sex × RT	1	189	1.74	0.1887
	Residuals	147	15 949		
Cell number ∼ Sex + RT	Sex	1	4 128 285	36.06	<0.001
	RT	1	4 128 207	36.06	<0.001
	Residuals	148	16 942 548		
Wing size ∼ Sex × RT	Sex	1	1.82 × 10^12^	800.92	<0.001
	RT	1	2.35 × 10^12^	1037.26	<0.001
	Sex × RT	1	1.51 × 10^10^	6.66	0.0108
	Residuals	147	3.33 × 10^11^		

### Thermal death time

**Fig. 3:**
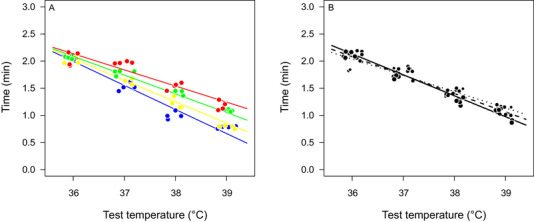
Thermal death time (*y*‐axis in log_10_ scale) curves of *Drosophila melanogaster* at different rearing temperatures and cell areas. (A) Thermal death time curves represent the prediction (solid lines) at the median cell area (filled circles) for different rearing temperatures (blue at 17 °C, yellow at 21 °C, green at 25 °C, and red at 29 °C). (B) Thermal death time curves represent predictions at rearing temperature of 25 °C for small (first quartile = 151 *µ*m^2^, dotted line), median (median = 177 *µ*m^2^, segmented line), and large (third quartile = 207 *µ*m^2^, solid line) cell area and represented by dots of different sizes for illustrative purposes. Plots were based on the model with the highest support for survival time shown in Table 4.

The temperature used during the heat stress trials strongly affected the survival times; mild heat stress could be tolerated for longer than extreme heat stress, reflecting the interplay between stress intensity and stress duration (Table 3). Curves describing the thermal death time did differ with rearing temperature (Fig. 3A), with flies reared at warmer conditions being better in surviving extreme heat stress. Because flies reared at warm conditions also had smaller cells, a better heat tolerance could also be directly related to differences in cell size. Cell area significantly affected the survival time when tested instead of rearing temperature, but explained less variation on survival time compared to a model based only in rearing temperature (Table 3). However, a model that included cell area, together with rearing temperature and stress temperature best explained variation in survival time, showing the highest support among all tested models (Table 3). This model indicated that flies with smaller cells were better at surviving acute exposure to intense heat, while this pattern reversed when flies were exposed to mild heat (Table 4 and Fig. 3B). Thermal death time curves did not differ significantly between males and females (Table 4), gaining less support than the model that only included stress temperature.

**Table 3:** Selection results of linear models to explain variation in the survival time of *Drosophila melanogaster* (in log_10_ scale) as a function of stress temperature (ST), rearing temperature (RT), cell area (CA) and sex

Effects	*k*	*R* ^2^_adj_	AICc	ΔAICc	*w_i_*	*LL*
**ST × RT + ST × CA**	**7**	**0.96**	**−113.07**	**0.00**	**0.35**	**64.54**
ST × RT	5	0.96	−113.01	0.06	0.34	62.02
ST × RT + ST × Sex	7	0.96	−112.44	0.63	0.26	64.22
ST × RT + ST × Sex + ST × CA	9	0.96	−108.47	4.50	0.04	64.95
ST × CA + ST × Sex	7	0.95	−105.45	7.62	0.01	60.73
ST × RT × CA × Sex	17	0.96	−92.82	20.25	0.00	70.06
ST × CA	5	0.91	−68.17	44.90	0.00	39.60
ST	3	0.82	−23.18	89.89	0.00	14.79
ST × Sex	5	0.82	−19.53	93.54	0.00	15.28

The number of parameters (*k*), the goodness of fit (*R* ^2^_adj_), corrected Akaike's information criterion (AICc), the difference in AICc value respect to the best model ΔAICc, Akaike's weights (*w_i_*), and log‐likelihood (*LL*) are indicated for each model. Models in bold show the ΔAICc < 2.

**Table 4:** Outcomes of linear models with the ΔAICc < 2 for the survival time of *Drosophila melanogaster* (in log_10_‐scale) highlighted in Table 3. Degrees of freedom (df), sum of squares (SS), Fisher (*F*) statistics, and probability values (*P*) are indicated for each model

Model	Source	df	SS	*F*	*P*‐value
Survival time ∼ ST × RT + ST × CA	ST	1	10.88	1265.79	<0.001
	RT	1	0.49	56.57	<0.001
	CA	1	0.00	0.51	0.4788
	ST × RT	1	0.10	11.75	0.0011
	ST × CA	1	0.03	4.23	0.0442
	Residuals	58	0.50		
Survival time ∼ ST × RT	ST	1	10.88	1210.50	<0.001
	RT	1	1.32	146.49	<0.001
	ST × RT	1	0.50	56.02	<0.001
	Residuals	60	0.54		
Survival time ∼ ST × RT + ST × Sex	ST	1	10.88	1253.42	<0.001
	RT	1	1.32	151.69	<0.001
	Sex	1	0.00	0.02	0.6944
	ST × RT	1	0.50	58.00	<0.001
	ST × Sex	1	0.03	3.97	0.0510
	Residuals	58	0.50		

**Fig. 4:**
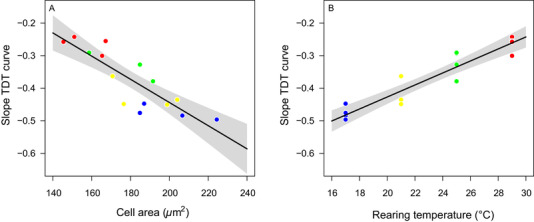
Slopes of the thermal death time (TDT) curves in relation to the cell size (A) and rearing temperature (B) for the 16 different treatments (2 densities × 2 sexes × 4 rearing temperatures). Rearing temperatures are color coded (blue at 17 °C, yellow at 21 °C, green at 25 °C, and red at 29 °C).

Both cell size (*R* ^2^_adj_ = 0.74, *P* < 0.001; Fig. 4A) and rearing temperature (*R* ^2^_adj_ = 0.88, *P* < 0.001; Fig. 4B) were correlated with the slope of the thermal death time curves. Flies with larger cells, or reared at lower temperatures, exhibited steeper negative slopes, meaning that their survival declined more rapidly when heat stress became more intense.

**Fig. 5:**
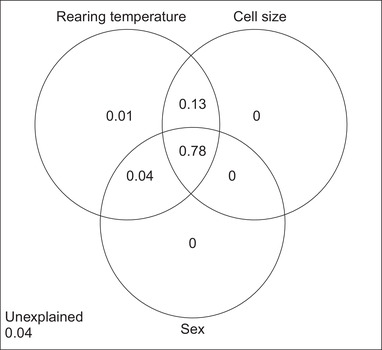
Variance from linear models partitioned into independent contributions of rearing temperature, sex, and cell area, as well as covariation among these variables to explain the survival time of *Drosophila melanogaster*.

Given the evident correlation between different explanatory variables (e.g., between rearing temperature and cell area), we partitioned the variance and found that most of the variation (78%) in thermal tolerance was explained by combined effects of rearing temperature, sex and cell size (Fig. 5). An additional 13% reflected combined effects of rearing temperature and cell size (i.e., independent of sex), and only 4% reflected combined effects of rearing temperature and sex (i.e., independent of cell size). The remaining variation was mostly unexplained (4%) or represented unique effects of rearing temperature alone (1%) (Fig. 5).

## Discussion

Rearing flies at different temperatures affected development time, wing size, cell area (as a proxy for cell size), and cell number (Fig. 2). Wing size, used here as a proxy of body size, decreased at warmer temperatures, which is consistent with the temperature‒size rule (Atkinson, 1996). Increases in wing size arose mainly by increases in cell size, rather than cell number (Table 2). Previous studies in *Drosophila melanogaster* females have likewise shown that increasing in body size under colder rearing temperature is a result of increase in cell size, whereas effects of food availability were manifested *via* changes in cell number (French *et al*., 1998; Arendt, 2007; Adrian *et al*., 2016).

We found clear differences in the survival time of *D. melanogaster* reared at different temperatures, with warm‐reared ones generally showing improved survival time (Fig. 4). Similar beneficial effects of a warmer thermal history, including heat hardening, have been reported by others for the same species (Sejerkilde *et al*., 2003; Cooper *et al*., 2012) and for ectotherms in general (Gunderson & Stillman, 2015). Because the impact of heat stress depends on both the intensity and duration of the thermal challenge, a single temperature (i.e., critical thermal maxima *CT*_max_) does not capture the entire thermal tolerance of a species (Rezende *et al*., 2014). By incorporating relationships between survival time at different temperatures such temporal aspects are included and reveal the thermal tolerance landscape. Indeed, our research showed that the effect of rearing temperature differed across this landscape. Effects of rearing temperature were more pronounced when flies had to tolerate extreme heat, but they became less influential at milder levels of heat stress (Figs. 2A and 4B). Previous studies that have characterized the thermal tolerance landscape also found variations in thermal death time curves, which could be related to ontogeny (Truebano *et al*., 2018), latitude (Castañeda *et al*., 2015), acclimation and rearing temperature (Castañeda *et al*., 2015; Semsar‐Kazerouni & Verberk, 2018), lineages (Paraskevopoulou *et al*., 2018) and interspecific differences (Jørgensen *et al*., 2019). This suggests that explicitly accounting for duration provides for a more complete picture of the drivers of heat tolerance (Leiva *et al*., 2019).

**Fig. 6:**
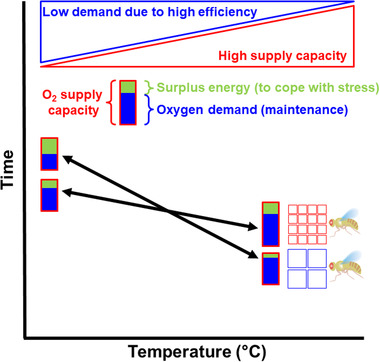
Schematic illustration of how differences in cell size are hypothesized to change the balance between oxygen supply and demand. The resulting differences in energy surplus (in green) have consequences for heat tolerance that play out differently for extreme heat (favoring small celled fruit flies with a higher supply capacity) and mild, but chronic heat stress (favoring large celled fruit flies with a higher efficiency).

Differences in heat tolerance can be explained by beneficial effects of thermal history, but also by differences in cell size, as rearing temperature affected cell size. Flies composed of smaller cells were better at surviving high‐stress temperature but worse at low‐stress temperature relative to flies composed of larger cells (Fig. 3B). At the cellular level, smaller cells with higher surface area to volume ratio have a larger capacity for supplying oxygen to the mitochondria (Szarski, 1983), which may prevent oxygen limitation at thermal extremes (Pörtner, 2001). Evidence for oxygen limiting thermal tolerance is weak in air‐breathing arthropods such as fruit flies (for a discussion, see Verberk *et al*., 2016), likely because of the effectiveness of the tracheal system in supplying oxygen. Manipulations of environmental oxygen have failed to demonstrate insufficient capacity of the tracheal system to supply oxygen during extreme heat under normoxia in *D. melanogaster* (Mölich *et al*., 2012), although heat tolerance is affected under more extreme levels of hypoxia (< 10 kPa; Lighton, 2007). However, the rate‐limiting step of oxygen delivery could lie in the diffusion of oxygen across the cell membranes, rather than the transport of oxygen by the tracheal system. A reduction in cell size would then increase the supply of oxygen by reducing the distance from the tracheole to mitochondria (Harrison *et al*., 2018), as well as increasing the membrane surface area to volume ratio, explaining why cell size is reduced when *D. melanogaster* is reared under mild hypoxia (Heinrich *et al*., 2011). Under this framework, flies composed of smaller cells could better meet the elevated oxygen demand at high temperatures than flies composed of larger cells (Fig. 6). At mild heat stress and longer time scales, efficiency may be at a premium, conferring an advantage to larger cells and their lower energy costs for maintaining electrochemical ionic gradients. Higher energetic efficiency can help to deal with the longer stress duration associated with mild heat stress. Thus, differences in cell size could provide a mechanism to explain why the effects of rearing temperature are more pronounced when flies had to tolerate extreme heat, but become less influential at mild levels of heat stress (Fig. 6).

Using the same framework, Castañeda *et al*. (2015) report survival times in the congener *Drosophila subobscura*, and they similarly found that flies reared at warmer temperatures had, on average, better heat tolerance. In contrast to our results, they report that the effects of rearing temperature became more influential at milder levels of heat stress (thermal death time curves diverged at milder levels of heat stress, opposite to our results). This difference between studies could be related to the focal species. One differences between the two species is that *D. subobscura* originates from temperate regions while *D. melanogaster* originates from tropical environments (Lachaise *et al*., 1988). This explains the lower heat tolerance of *D. subobscura*, and similar differences in thermal tolerance between both species are also reported by Jørgensen *et al*. (2019), and the parameters derived from the thermal death time curves, *CT*_max_ and *z*‐values, are comparable with those found in our study (Table S5). Still, it is not clear how this difference in thermal tolerance would result in rearing temperature having opposite effects on the thermal death time curves of the two species (thermal death time curves diverged at milder levels of heat stress in *D. subobscura*, while they converged in our results on *D. melanogaster*). Another possibility is that rearing temperature did not (greatly) affect cell size of the *D. subobscura* populations used by Castañeda *et al*. (2015). There is some evidence that this may be the case for the South American populations studied by Castañeda and colleagues. Calboli *et al*. (2003) studied latitudinal clines in body size and cell size in *D. subobscura* for both Europe, North and South America populations and report that the latter ones show latitudinal clines in body size that are based on differences in cell number, but not in cell size. If for *D. subobscura* differences in rearing temperature did not induce contrasts in cell size, the mechanism suggested for our study species (Fig. 6) would not operate, providing an explanation for why rearing temperature affected the thermal death time curves differently.

In our research, rearing temperatures and cell size were correlated and thus the unique effects of each one are difficult to disentangle. Temperature affects not just cell size, but also modulates a range of other phenotypic traits, including membrane fluidity *via* homeoviscous adaptation and enzyme activity *via* expressions of isozymes and heat shock proteins, all of which are known to also play a role in surviving heat stress (Stillman, 2002; Van der Have, 2002). Additionally, there is evidence that many intracellular processes scale with cell size, which may also contribute to variations in protein concentration and mitochondrial functions that, as a whole, could affect the biochemical reactions of the cells (Amodeo & Skotheim, 2016; Miettinen & Björklund, 2017). Yet, 91% of the variation in heat tolerance reflected shared variation explained by both cell size and rearing temperature (Fig. 5), while only 5% could be ascribed to effects of rearing temperature that were independent of cell size. This suggests that cell size is at least potentially an important factor in explaining the differences in thermal tolerance among flies reared at different temperatures. The suggestion that warm rearing temperatures lead to reductions in cell size and that such reductions are adaptive as small cells can better meet elevated demands for oxygen during heat exposure requires further research. Researchers have begun addressing different aspects, showing concordant effects of temperature on the body size and cell area of various ectotherms (Czarnołęski *et al*., 2015; Walczyńska *et al*., 2015; Czarnołęski *et al*., 2017; Kierat *et al*., 2017), demonstrating greater thermal effects on body size under low oxygen (Frazier *et al*., 2001; Hoefnagel & Verberk, 2015), and that small‐celled organisms perform better under low oxygen levels, while large‐celled organisms perform better under high oxygen levels (Walczyńska *et al*., 2015).

In conclusion, flies reared at warmer temperatures have smaller cells, and are better at surviving thermal extremes. Flies reared at lower temperatures having larger cells and are better at surviving milder levels of heat stress. Changes in cell size could be the cause for these differences, with small cells conferring increased tolerance to extreme heat via enhanced oxygen delivery to their mitochondria and large cells conferring increased tolerance to mild heat stress *via* more efficient use of resources. Future studies manipulating environmental oxygen availability could test this idea. Here, rearing temperature was used to induce contrasts in cell size, which confounds the effects of cell size on thermal tolerance. A plausible alternative is the use of different stocks of isogenic fruit flies that exhibit contrasting cell sizes, which may improve our understanding of thermal responses from a cell size perspective.

## Disclosure

The authors declare that they have no competing interests.

## Data Accessibility

Data files used in this study are available on the DANS EASY data repository (https://doi.org/10.17026/dansz2a-9m4g).

## Supporting information


**Table S1**. Selection results of linear models to explain variation in the cell area, cell number, and wing size in*Drosophila melanogaster* as a function of sex, rearing density (RD) and rearing temperature (RT). The number of parameters (*k*), the goodness of fit (*R* ^2^_adj_), corrected Akaike's information criterion (AICc), the difference in AICc value respect to the best model ΔAICc, Akaike's weights (*w_i_*) and log‐likelihood (*LL*) are indicated for each model. Bold indicates models with the ΔAICc < 2.
**Table S2**. Outcomes of linear models with the ΔAICc < 2 for cell area, cell number and wing size highlighted in Table S3. Degrees of freedom (df), sum of squares (SS), Fisher statistic (*F*), and probability values (*P*) are indicated for each model.
**Table S3**. Selection results of linear models to explain variation in the survival time of *Drosophila melanogaster* (in log_10_‐scale) as a function stress temperature (ST), rearing temperature (RT), cell area (CA), and sex. Models were compared using the mean or the median of cell area. The number of parameters (*k*), the goodness of fit (*R* ^2^_adj_), corrected Akaike's information criterion (AICc), and log‐likelihood (*LL*) are indicated for each model.
**Table S4**. Selection results of linear models to explain variation in wing size in *Drosophila melanogaster* as a function of cell size and cell number. The number of parameters (*k*), the goodness of fit (*R* ^2^_adj_), corrected Akaike's information criterion (AICc), Akaike's weights (*w_i_*), and log‐likelihood (*LL*) are indicated for each model. Bold indicates models with the highest support.
**Table S5**. Heat tolerance parameters derived from thermal death time (TDT) curves described by the model with the highest support presented in Table 3. Static critical thermal maxima (CT_max_) at 1 min were derived from semilog linear regressions performed between survival time, in log‐scale, against the static assayed temperature.
**Fig. S1**. Effects of rearing temperature and rearing density in cell number of *Drosophila melanogaster*. Predicted effects (lines) and 95% confidence intervals (light shaded areas) are based upon the regression model for cell number showed in Table 2, which shows interactive effects of rearing density and rearing temperature (RD × RT; *P* = 0.0057).
