# Supplementary material for: Effects of developmental plasticity on heat tolerance may be mediated by changes in cell size in Drosophila melanogaster
Source: Insect Sci. 2020 Jan 17;27(6):1244–56. doi: 10.1111/1744-7917.12742 (PMC7687148; doi:10.1111/1744-7917.12742)
Supplement: Supplementary file 1 — Table S1. Selection results of linear models to explain variation in the cell area, cell number, and wing size inDrosophila melanogaster as a function of sex, rearing density (RD) and rearing temperature (RT). The number of parameters (k), the goodness of fit (R 2 adj), corrected Akaike's information criterion (AICc), the difference in AICc value respect to the best model ΔAICc, Akaike's weights (wi) and log‐likelihood (LL) are indicated for each model. Bold indicates models with the ΔAICc < 2. Table S2. Outcomes of linear models with the ΔAICc < 2 for cell area, cell number and wing size highlighted in Table S3. Degrees of freedom (df), sum of squares (SS), Fisher statistic (F), and probability values (P) are indicated for each model. Table S3. Selection results of linear models to explain variation in the survival time of Drosophila melanogaster (in log10‐scale) as a function stress temperature (ST), rearing temperature (RT), cell area (CA), and sex. Models were compared using the mean or the median of cell area. The number of parameters (k), the goodness of fit (R 2 adj), corrected Akaike's information criterion (AICc), and log‐likelihood (LL) are indicated for each model. Table S4. Selection results of linear models to explain variation in wing size in Drosophila melanogaster as a function of cell size and cell number. The number of parameters (k), the goodness of fit (R 2 adj), corrected Akaike's information criterion (AICc), Akaike's weights (wi), and log‐likelihood (LL) are indicated for each model. Bold indicates models with the highest support. Table S5. Heat tolerance parameters derived from thermal death time (TDT) curves described by the model with the highest support presented in Table 3. Static critical thermal maxima (CTmax) at 1 min were derived from semilog linear regressions performed between survival time, in log‐scale, against the static assayed temperature. Fig. S1. Effects of rearing temperature and rearing density in cell number of Drosophila [file INS-27-1244-s001.docx]

**SUPPORTING INFORMATION**

**Effects of developmental plasticity on heat tolerance may be mediated by changes in cell size in *Drosophila melanogaster***

Nadja Verspagen^a^, Félix P. Leiva^a,*^, Irene Janssen^b^, Wilco C.E.P. Verberk^a^

^a^ Department of Animal Ecology and Physiology, Institute for Water and Wetland Research, Radboud University, Nijmegen, The Netherlands.

^b^ Department of Human Genetics, Radboud Institute for Molecular Life Sciences, Radboud University Medical Center, Nijmegen, The Netherlands.

*Author for correspondence: Félix P. Leiva; Department of Animal Ecology and Physiology, Institute for Water and Wetland Research, Radboud University, P.O. Box 9010, 6500 GL Nijmegen, The Netherlands; f.leiva@science.ru.nl; Tel. +31 24 36 52617; ORCID ID: 0000-0003-0249-9274.

Table S1. Selection results of linear models to explain variation in the cell area, cell number and wing size in *Drosophila melanogaster* as a function of sex, rearing density (RD) and rearing temperature (RT). The number of parameters (*k*), the goodness of fit (*R*^2^_adj_), corrected Akaike’s information criterion (AICc), the difference in AICc value respect to the best model ΔAICc, Akaike’s weights (*w_i_*) and log-likelihood (*LL*) are indicated for each model. Bold indicates models with the ΔAICc < 2.

| **Response** | **Effects** | ***k*** | ***R*^2^_adj_** | **AICc** | **ΔAICc** | ***w_i_*** | ***LL*** |
| --- | --- | --- | --- | --- | --- | --- | --- |
| Cell area | **Sex + RT** | 4 | 0.80 | 1142.22 | **0.00** | 0.45 | -566.97 |
|  | **Sex + RD × RT** | 6 | 0.80 | 1142.62 | **0.40** | 0.36 | -565.02 |
|  | Sex + RD + RT | 5 | 0.79 | 1144.35 | 2.13 | 0.15 | -566.97 |
|  | Sex × RD × RT | 9 | 0.80 | 1147.29 | 5.08 | 0.04 | -564.01 |
|  | RT | 3 | 0.46 | 1287.31 | 145.09 | 0.00 | -640.57 |
|  | RD + RT | 4 | 0.46 | 1289.35 | 147.13 | 0.00 | -640.54 |
|  | Sex | 3 | 0.32 | 1322.11 | 179.89 | 0.00 | -657.97 |
|  | RD + Sex | 4 | 0.32 | 1324.14 | 181.93 | 0.00 | -657.93 |
|  | RD | 3 | 0.00 | 1382.39 | 240.17 | 0.00 | -688.11 |
| Cell number | **Sex + RD × RT** | 6 | 0.36 | 2185.47 | **0.00** | 0.88 | -1086.45 |
|  | Sex + RT+ RD | 5 | 0.33 | 2191.23 | 5.75 | 0.05 | -1090.41 |
|  | Sex × RD × RT | 9 | 0.35 | 2191.46 | 5.98 | 0.04 | -1086.09 |
|  | Sex + RT | 4 | 0.32 | 2192.63 | 7.16 | 0.02 | -1092.18 |
|  | RD + RT | 4 | 0.17 | 2222.07 | 36.60 | 0.00 | -1106.90 |
|  | RD + Sex | 4 | 0.17 | 2223.11 | 37.63 | 0.00 | -1107.42 |
|  | RT | 3 | 0.16 | 2223.45 | 37.97 | 0.00 | -1108.64 |
|  | Sex | 3 | 0.16 | 2223.45 | 37.97 | 0.00 | -1108.64 |
|  | RD | 3 | 0.01 | 2247.75 | 62.28 | 0.00 | -1120.79 |
| Wing size | **Sex × RD × RT** | 9 | 0.93 | 3688.54 | **0.00** | 0.62 | -1834.63 |
|  | Sex + RT + RD | 5 | 0.92 | 3690.74 | 2.19 | 0.21 | -1840.16 |
|  | Sex + RT | 4 | 0.92 | 3692.34 | 3.80 | 0.09 | -1842.03 |
|  | Sex + RD × RT | 6 | 0.92 | 3692.46 | 3.91 | 0.09 | -1839.94 |
|  | RT | 3 | 0.52 | 3966.04 | 277.49 | 0.00 | -1979.94 |
|  | RD + RT | 4 | 0.52 | 3966.84 | 278.29 | 0.00 | -1979.28 |
|  | Sex | 3 | 0.40 | 3999.44 | 310.90 | 0.00 | -1996.54 |
|  | RD + Sex | 4 | 0.39 | 4001.36 | 312.81 | 0.00 | -1996.54 |
|  | RD | 3 | 0.00 | 4076.29 | 387.75 | 0.00 | -2035.06 |

Table S2. Outcomes of linear models with the ΔAICc < 2 for cell area, cell number and wing size highlighted in Table S3. Degrees of freedom (d.f.), Sum of squares (SS), Fisher statistic (*F*) and probability values (*P*) are indicated for each model.

| **Model** | **Source** | **d.f.** | **SS** | ***F*** | ***P*-value** |
| --- | --- | --- | --- | --- | --- |
| Cell area ~ Sex + RT | Sex | 1 | 26641 | 244.32 | < 0.001 |
|  | RT | 1 | 37728 | 345.99 | < 0.001 |
|  | Residuals | 148 | 16138 |  |  |
| Cell area ~ Sex + RD × RT | Sex | 1 | 26431 | 245.38 | < 0.001 |
|  | RD | 1 | 27 | 0.25 | 0.6193 |
|  | RT | 1 | 37702 | 350.02 | < 0.001 |
|  | RD × RT | 1 | 411 | 3.82 | 0.0526 |
|  | Residuals | 146 | 15726 |  |  |
| Cell number ~ Sex + RD × RT | Sex | 1 | 4442831 | 38.52 | < 0.001 |
|  | RD | 1 | 393112 | 3.65 | 0.0578 |
|  | RT | 1 | 4182060 | 38.88 | < 0.001 |
|  | RD × RT | 1 | 845636 | 7.86 | 0.0057 |
|  | Residuals | 146 | 15703800 |  |  |
| Wing size ~ Sex × RD × RT | Sex | 1 | 1.80 × 10^12^ | 817.96 | < 0.001 |
|  | RD | 1 | 7.90 × 10^9^ | 3.58 | 0.0606 |
|  | RT | 1 | 2.35 × 10^12^ | 1062.75 | < 0.001 |
|  | Sex × RD | 1 | 7.89 × 10^9^ | 3.57 | 0.0609 |
|  | Sex × RT | 1 | 1.54 × 10^10^ | 6.96 | < 0.001 |
|  | RD × RT | 1 | 1.46 × 10^9^ | 0.66 | 0.4178 |
|  | Sex × RD × RT | 1 | 1.87 × 10^8^ | 0.08 | 0.7715 |
|  | Residuals | 143 | 3.16 × 10^11^ |  |  |

Table S3. Selection results of linear models to explain variation in the survival time of *Drosophila melanogaster* (in log_10_-scale) as a function stress temperature (ST), rearing temperature (RT), cell area (CA) and sex. Models were compared using the mean or the median of cell area. The number of parameters (*k*), the goodness of fit (*R*^2^_adj_), corrected Akaike’s information criterion (AICc) and log-likelihood (*LL*) are indicated for each model.

| **Models for survival time** | ***R*^2^_adj_** | | **AICc** | | ***LL*** | |
| --- | --- | --- | --- | --- | --- | --- |
|  | **mean** | **median** | **mean** | **median** | **mean** | **median** |
| ST × RT + ST × CA (*k* = 7) | 0.96 | 0.96 | -112.45 | -113.07 | 64.23 | 64.54 |
| ST × RT + ST × Sex + ST× CA (*k* = 9) | 0.96 | 0.96 | -107.97 | -108.47 | 64.65 | 64.95 |
| ST × CA + ST × Sex (*k* = 7) | 0.96 | 0.95 | -108.27 | -105.45 | 62.14 | 60.73 |
| ST × RT × CA × Sex (*k* = 17) | 0.96 | 0.96 | -91.83 | -92.82 | 69.57 | 70.06 |
| ST × CA (*k* = 5) | 0.91 | 0.91 | -67.61 | -68.17 | 39.32 | 39.60 |

Table S4. Selection results of linear models to explain variation in wing size in *Drosophila melanogaster* as a function of cell size and cell number. The number of parameters (*k*), the goodness of fit (*R*^2^_adj_), corrected Akaike’s information criterion (AICc), Akaike’s weights (*w_i_*) and log-likelihood (*LL*) are indicated for each model. Bold indicates models with the highest support.

| **Model** | ***k*** | ***R*^2^_adj_** | **AICc** | ***w_i_*** | | ***LL*** |
| --- | --- | --- | --- | --- | --- | --- |
| **Wing size ~ Cell area + Cell number** | 4 | 0.99 | 3170.95 | **1.00** | -1581.34 | |
| Wing size ~ Cell area | 3 | 0.84 | 3801.84 | 0.00 | -1897.84 | |
| Wing size ~ Cell number | 3 | 0.38 | 4003.33 | 0.00 | -1998.58 | |

Table S5. Heat tolerance parameters derived from thermal death time (TDT) curves described by the model with the highest support presented in Table 3. Static critical thermal maxima (CTmax) at 1 minute were derived from semi-log linear regressions performed between survival time, in log-scale, against the static assayed temperature.

| **Rearing temperature (°C)** | **Cell area (µm^2^)** | ***z-value*** | **CTmax (°C)** |
| --- | --- | --- | --- |
| 17 | 151 | 2.51 | 40.89 |
| 17 | 177 | 2.28 | 40.54 |
| 17 | 207 | 2.07 | 40.22 |
| 21 | 151 | 2.86 | 41.70 |
| 21 | 177 | 2.57 | 41.23 |
| 21 | 207 | 2.30 | 40.78 |
| 25 | 151 | 3.33 | 42.79 |
| 25 | 177 | 2.94 | 42.12 |
| 25 | 207 | 2.59 | 41.52 |
| 29 | 151 | 3.96 | 44.29 |
| 29 | 177 | 3.43 | 43.30 |
| 29 | 207 | 2.96 | 42.96 |


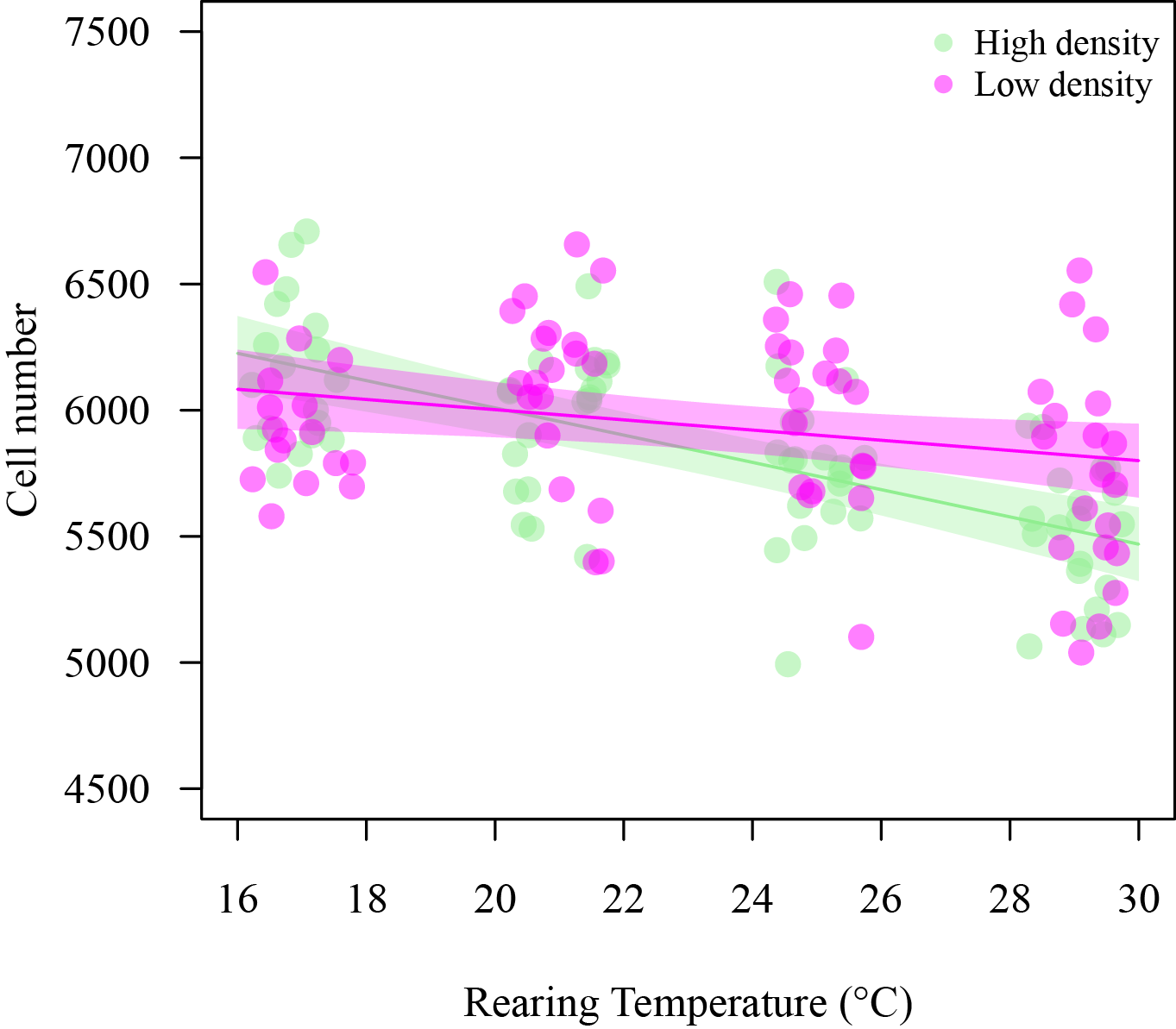


Fig. S1. Effects of rearing temperature and rearing density in cell number of *Drosophila melanogaster.* Predicted effects (lines) and 95% confidence intervals (light shaded areas) are based upon the regression model for cell number showed in Table 2 (main text), which shows interactive effects of rearing density and rearing temperature (RD × RT; *P* = 0.0057).
